# Public perceptions of the Hippocratic Oath in the U.K. 2023

**DOI:** 10.1186/s12910-024-01127-8

**Published:** 2024-11-04

**Authors:** Ben Green

**Affiliations:** The Medical School, University of Central Lancashire, Preston, Lancashire U.K.

**Keywords:** Oath, Hippocratic, Public, Ethics, Principlism

## Abstract

This paper explores public perceptions of the Hippocratic Oath (Physician’s Oath) in the U.K. Results of a questionnaire administered online to 106 adults indicated that the majority were of the opinion that their primary and secondary health care doctors had taken the Oath (88% and 86% respectively). A majority thought that nurses, paramedics, psychotherapists and graduate scientists and researchers should also take some form of professional oath. Elements of the Oath which were deemed most important included that it is a sworn oath, that doctors should not harm patients, act in the best interests of patients, abide by the principles of autonomy and informed consent and maintain patient confidentiality. A significant proportion - about 20% - of the UK public felt that doctors had forgotten their Hippocratic Oath during COVID-19 lockdowns and associated vaccination programme, suggesting that recent history may have damaged the public faith in the medical profession.

## Introduction

The Hippocratic Oath, the medical professions’ ethical oath, has enjoyed something of a resurgence in UK medical education with 70% of UK medical schools reporting that they now use the Oath [1]. Research on how UK universities perceive the Oath showed that they often use the Oath on admission to and graduation from medical school and the schools saw the Oath to signify transition into the medical profession and to promote professional values affecting patient care [1].

The Hippocratic Oath reflects a prevailing ethos and has been characterised as the cornerstone of the medical profession, providing a synopsis of Ancient Greek medicine, enshrining the tripartite relationship between doctor, patient and illness [2]. The oath used in the western world has evolved from the original and differs with regard to issues such as abortion, euthanasia and autonomy [2]. Antoniou et al. were concerned that technological evolution, public media, and the drive for cost-effectiveness would disrupt this relationship [2], and that the relationship between doctor and patient needed reinstatement.

This paper concerns how the Oath is perceived from the perspective of the UK public and for this purpose an all-new 11-item questionnaire was administered to a randomised online panel of over 100 adults.

An extensive database search indicated that this is new research without any previously published equivalent.

In addition, enquiries were made to the UK medical regulator, the General Medical Council, regarding their position regarding the Oath and UK doctors.

## Method

A 11-item questionnaire was administered to a randomised, mixed group of over 150 UK adults (18 or over) using an online survey site. The random sample approached reflected the UK population in terms of age and sex.

Hypotheses about how the public might perceive the Hippocratic Oath generated a pilot questionnaire which was then discussed in a focus group. The focus group raised additional issues such as their perception that doctors had not followed the Oath during the recent pandemic and lockdown. Additional questions were formulated and original questions adjusted to form a final questionnaire was formatted on and distributed via a proprietary online survey platform, which includes features to ensure the security and privacy of collected data, including encryption and compliance with data protection standards. The system allowed a randomised, age and sex stratified sample to match that of the general adult population in the UK. Ethical approval for the research was sought and obtained under University guidelines. A copy of the Hippocratic oath was supplied for reference and the following 11 questions were asked:

1. Have you ever heard of bioethics or bioethical principles Yes/No
2. Do you think your regular primary care doctor has taken the Hippocratic Oath? Yes/No/Don’t Know
3. Do you think the last hospital doctor you saw took the Hippocratic Oath? Yes / No/ Don’t Know
4. Which of the following health professionals should also take a form of the Hippocratic Oath?

The following non-exclusive choices were given – Nurses / Paramedics / Psychotherapists and counsellors / Occupational Therapists / Health administrators / Hospital Porters / None of the above

5. Which do you think are the important parts of the original Hippocratic Oath for doctors (tick which you personally think are important)?

The following non-exclusive choices were given – That it is a sworn oath / Respecting those who teach medicine / Training other doctors / Prescribing the right diet / Ensuring patients are not harmed / Not administering poisons / Not performing abortions / Being truthful / Not sexually abusing a patient / Maintaining patient confidentiality / None of the above.

6. Should the Hippocratic Oath stay the same or should it change and reflect current society?

The following choices were given :

Stay the same / Change to reflect society / Don’t Know

7. If there was a new Hippocratic Oath what elements should it include?

The following non-exclusive choices were given – Do no harm to the patient / Do not allow financial inducements / Declare any financial interests / Always act in the best interests of the patient / Always seek informed consent / Put medical ethics ahead of the law or current trends / None of the above.

8. When should doctors take the Oath?

The following non-exclusive choices were given – When they enter medical school / When they graduate as doctors/ Renew it every year / Never

9. Should graduate scientists and researchers also take an oath to behave ethically?

Yes /No / Don’t Know

10. Do you think doctors forgot their Hippocratic Oath during the Covid- 19 lockdowns?

Yes /No / Don’t Know

A free text box allowed responders to explain their answer.

11. Do you think doctors forgot their Hippocratic Oath during the Covid- 19 vaccination programs?

Yes /No / Don’t Know

A free text box allowed responders to explain their answer.

## Results

Out of 152 UK adults, there was a response rate of 70%. Of the 106 respondents 49% were female and 51% were male. 17% were aged 18–29, 30% were aged 30–44, 34% were aged 45–60, and 19% were aged over 60.

Responses to the question ‘Have you ever heard of bioethics or bioethical principles?’ indicated that most respondents (*n* = 56, 53%) had some familiarity with these terms.

Responses to the question ‘Do you think your regular primary care doctor has taken the Hippocratic Oath?’ indicated that most respondents (*n* = 93, 87.7%) believed their primary care doctor had taken the Hippocratic Oath. Only six (5.7%) thought their primary care doctor had not taken the Oath and seven (6.6%) did not know.

Responses to the question ‘Do you think the last hospital doctor you saw took the Hippocratic Oath?’ indicated that most respondents (*n* = 91, 85.9%) believed their hospital doctor had taken the Hippocratic Oath. Only five (4.7%) thought their primary care doctor had not taken the Oath and ten (9.4%) did not know.

Responses to the question ‘Which other health professionals should take a form of the Hippocratic oath?’ (see Table 1) indicated that most respondents thought nurses, paramedics, psychotherapists and counsellors should also take a form of the Hippocratic Oath. Only a minority thought that occupational therapists, health administrators and hospital porters should take an ethical oath. !7% of respondents thought no other professionals should take a form of a Hippocratic Oath. Sex did not affect the answers to this question.

**Table 1:** Which other health professionals should take a form of the Hippocratic Oath?

Professional group	Respondents (*n*=106)	Percentage
Nurses	71	66.98%
Paramedics	66	62.26%
Psychotherapists and counsellors	58	54.72%
Occupational therapists	41	38.68%
Health administrators	32	30.19%
Hospital Porters	20	18.87%
None of the above	18	16.98%

Members of the public were asked to consider the original Hippocratic Oath (please see Table 2) and highlight which elements they considered important. Elements they were asked to consider included; that it is a sworn oath, that doctors should respect their teachers, that doctors should engage to train other doctors, prescribe the right diet, not administer poison, not perform abortions, be truthful, ensure patients are not harmed, not have affairs with patients, and maintain patient confidentiality. Elements that were most often voted important included that the oath is solemnly sworn by doctors, that they ensure no harm comes to patients, maintain patient confidentiality and are truthful. Only 9 respondents (8.49%) thought it important that doctors not perform abortions (See Table 3). Sex did not affect the answers to the question.

**Table 2:** The original text of the Hippocratic Oath

“I swear by Apollo the physician, and Aesculapius the surgeon, likewise Hygeia and Panacea, and call all the gods and goddesses to witness, that I will observe and keep this underwritten oath, to the utmost of my power and judgment.
I will reverence my master who taught me the art. Equally with my parents, will I allow him things necessary for his support, and will consider his sons as brothers. I will teach them my art without reward or agreement; and I will impart all my acquirement, instructions, and whatever I know, to my master’s children, as to my own; and likewise to all my pupils, who shall bind and tie themselves by a professional oath, but to none else.
With regard to healing the sick, I will devise and order for them the best diet, according to my judgment and means; and I will take care that they suffer no hurt or damage.
Nor shall any man’s entreaty prevail upon me to administer poison to anyone; neither will I counsel any man to do so. Moreover, I will give no sort of medicine to any pregnant woman, with a view to destroy the child.
Further, I will comport myself and use my knowledge in a godly manner.
I will not cut for the stone, but will commit that affair entirely to the surgeons.
Whatsoever house I may enter, my visit shall be for the convenience and advantage of the patient; and I will willingly refrain from doing any injury or wrong from falsehood, and (in an especial manner) from acts of an amorous nature, whatever may be the rank of those who it may be my duty to cure, whether mistress or servant, bond or free.
Whatever, in the course of my practice, I may see or hear (even when not invited), whatever I may happen to obtain knowledge of, if it be not proper to repeat it, I will keep sacred and secret within my own breast.
If I faithfully observe this oath, may I thrive and prosper in my fortune and profession, and live in the estimation of posterity; or on breach thereof, may the reverse be my fate!”

**Table 3:** What are the important parts of the original Hippocratic Oath for doctors?

Element of the Oath	Respondents (*n*=106)	Percentage
That it is a sworn oath	66	62.26%
Ensuring patients are not harmed	62	58.49%
Maintaining patient confidentiality	54	50.94%
Being truthful	48	45.28%
Not sexually abusing a patient	36	33.96%
Respecting their teachers	34	32.08%
Not administering poisons	30	28.30%
Training other doctors	24	22.64%
Prescribing the right diet	13	12.26%
Not performing abortions	9	8.49%

Members of the public were asked whether the Oath should always remain the same or whether it should change to reflect changes in society. Most said it should stay the same (*n* = 48, 45.3%). 39 (36.8%) said it should evolve to reflect changes, and 19 (17.9%) said they did not know.

The next set of questions was based upon the revised Hippocratic Oath, based on bioethical principles, first published in 2017, reproduced in the Table 4 below.

**Table 4:** Modern Hippocratic Oath based on Bioethical principles (Green, 2017)

I declare that, as a foundation of my actions, I will practise my profession to the best of my knowledge, ability and insight, in good conscience and with probity, treating all people equally and fairly, without prejudice.
I will always remember my position of power and trust, and hold myself accountable for my actions and their consequences, eschewing recklessness.
I will respect the autonomy, confidences and dignity of all my patients in their living and in their dying.
In my practice the care and treatment of patients will be my first consideration. I will always seek to improve and maintain my patients’ health and strive to cause no deliberate or negligent harm to my patients or others.
I will strive to prevent and treat disease, improve quality of life, provide support in times of suffering and promote and protect the health and wellbeing of the communities that I live and work in.
I will treat my colleagues and all who contribute to the well being of my patients with respect.
I will continue to seek knowledge, understanding, and insight, to improve my clinical skills and to teach the art and science of medicine to others, as my teachers have done before me.
I will not breach these obligations, or abuse the trust placed in me, either under threat or for personal gain.
I make this declaration solemnly, freely, and in good faith.

The respondents were asked which would be the most important parts of the new Oath and the requirement to do no harm to the patient was mentioned by 62 (58.49%), to always act in the interests of the patient by 62 (58.49%), the requirement to abide by the principles of patient autonomy and informed consent was mentioned by 55 (51.89%), and to not allow financial inducements or threats to affect patient care by 45 (42.45%).

The respondents were asked when they thought doctors should take the Oath. 51 (48.11%) said it should be when they enter medical school, but most said when they graduate as doctors (61, 57.55%). 46 (43.40%) said that doctors should renew their oath every year.

A question on whether an oath to behave ethically should be extended to ‘graduate scientists and researchers’ was answered positively by 87 (82%) of respondents.

Most of the public (83, 78.30%) thought that doctors had not forgotten their Hippocratic Oath during the COVID-19 lockdowns. Reasons for this opinion were sought and the free text answers recorded included the following: “GPs hid behind the pandemic and continue to do so after it is over. Failing to see patients face to face and pushing their responsibilities on pharmacists, nurses and non-trained HCAs is putting lives at risk”, “They did not treat people who refused the vaccine fairly”, “Disrespect for patients”, “They wouldn’t see me”, “They pushed an unsupported narrative”, “They didn’t put patients first during this period”, “It was inhumane”, “Many people given DNR to save beds” and “they didn’t do their best to save all lives, but made choices that led to some people dying”.

A second question sought to separate out the experience of medical care during lockdowns and the vaccination programme, asking ‘Do you think doctors forgot their Hippocratic Oath during the COVID-19 vaccination programme?’ Again, most of the public (*n* = 86, 81.13%) thought that doctors had not forgotten their Hippocratic Oath during the COVID-19 lockdowns. However, a proportion (*n* = 20, 18.97%) thought they had forgotten their oath. Reasons for this opinion were sought and free text answers included: “They administered vaccines that weren’t totally safe”, “Pushing a non-properly tested vaccine is wrong”, “They put money over health”, “Disrespect for their patients”, “Pushed an untested vaccine”, “They didn’t know if it would do harm or not”.

## Conclusions

Although 70% of UK medical schools use the Oath [1], 85% of the general public believes that their own primary care and hospital doctors have taken the Oath. There is therefore something of a mismatch between the expectations held by the public of their doctors and reality. That same public might be surprised that the General Medical Council (GMC), the body that regulates doctors in the UK, only concerns itself with a code of medical practice called ‘Good Medical Practice’. As the Oath is not enshrined in statutory legislation the GMC does not take any position with regard to doctors taking the Hippocratic Oath and taking the Oath is not a condition of obtaining a licence to practice. Indeed, the GMC does not carry any data on whether any doctor has taken the Oath.

Does the taking of an Oath promote socially desirable professional behaviours and prevent unprofessional behaviours? One might also ask whether the locus of control of medical behaviours is internally determined or externally controlled. A public that might have faith in the deontological nature of the Oath, believe all doctors take the Oath, and that doctors weigh their medical decisions against basic ethical principles might be surprised by the reality that not all doctors take the Oath and that Regulators such as the GMC do not record whether the Oath is taken or refer to it, instead relying on a externalised list of published standards called *Good Medical Practice*, a document which does not include the words *Hippocratic*,* oath*,* ethic*, or *ethics* in its 36 pages [3].

Such an approach, focussing on acceptable actions and standards, is consequentialist, rather than deontological. The aspiration of applying principlism to practical ethics in medicine, according to Hain & Saad, would be to mitigate against, and avoid deception or coercion as exemplified by medical experimentation during World War II. They advocate that doctors weigh all their moral decisions afresh, reasoning from ethical principles, rather than merely allying their behaviour to external standards [4].

Most members of the public believed the Oath should be taken at graduation with a substantial proportion saying the Oath should be renewed every year, perhaps at their annual appraisal.

Most of this sample of the public thought a form of a bioethical oath should be extended to nurses, paramedics, psychotherapists and counsellors. Although there was only minority support for medical administrators, these are now individuals with a lot of power over a patient’s journey through healthcare settings, and a specific Oath for Chief Executives has been proposed by Borden [5].

There was also, surprisingly, an appetite for an ethical oath to be sworn by all graduate scientists and researchers with this idea finding favour with at least 82% of respondents. This is interesting as the fruits of scientific endeavour can be turned to antisocial ends with great potential for loss of human life – one immediately thinks of weapons development, gain of function research on pathogens, and errors in epidemiological modelling [6] – and wonders if a greater premium should be placed on scientific ethics at University. An ethical oath at graduation may be valuable in science [7]. Indeed, there has been a proposal for such an oath or an ethical code in mathematics [8]. Neuroscience, at a key point in its research on defining neurotechnologies that might achieve analysis of thought through neuronal data and achieve the targeted alteration of brain activity, is asking itself whether a ‘Technocratic Oath’ is required [9]. Given the potential for altering public perception of medicinal products there have been calls for an ethical oath for medical communicators, to ensure that health information is faithfully represented [10]. France has introduced an ‘integrity oath’ for PhD students including the pledge to “the greatest of my ability, to continue to maintain integrity in my relationship to knowledge, to my methods and to my results.” The Integrity Oath is taken at the start of the doctorate and when the PhD is conferred [11].

The responses to questions about doctors’ ethical behaviour during the years of COVID lockdown and vaccination programs mostly supported the medical profession, but there was a surprisingly high proportion of the public who felt that doctors had forgotten their oath during lockdowns (21.70%) and the vaccination programme (18.87%). This seems a worryingly high level of disapprobation for a usually highly trusted profession and may signal that considerable work will be needed to restore public faith in the ethics of the medical profession. Further research is indicated to replicate the findings and also to define any reasons why people felt doctors had abandoned the oath during the pandemic and associated vaccination programme and to consider whether there needs to be a reconfiguration of the oath, a re-evaluation of professional regulators or development of a professional culture that fosters and supports individual ethical and critical thinking.

Moukaddam and Tucci (2021) wrote about the tension that arose during the COVID pandemic, between medical ethics to put the individual first – for example to help dying patients say goodbye to their loved ones – and the adjurations or demands of healthcare and its mechanistic protocols [12]. Their contention was that one should ask “Is my loved one a client or patient? I want them to be a patient, not a client, a commodity that pays the bills. Healthcare has no heart and no allegiance. Medicine does. Medicine sees my loved one as a patient, to whom care, dedication, and sacrifice are due, and in this time of suffering, this is what we need, the moral shackles of the Hippocratic Oath and the Nightingale Pledge.” Their language is contentious, but undoubtedly heartfelt. Is the object of such oaths to promote ethical reasoning or, like the promoted standards of regulators, control behaviour? Further research is suggested to explore whether there is significant difference between the expectations of the public and the regulators with regard to how the profession makes ethical decisions - according to an internalised ethical framework and independent critical thinking or the imposition of an externally administered, third party authored, code of practice.

## Data Availability

The dataset generated and analysed during the current study is not publicly available for reasons of confidentiality but elements are available from the corresponding author on reasonable request.

## Abbreviations

GMC: General Medical Council
